# RNA Regulatory Networks 2.0

**DOI:** 10.3390/ijms24109001

**Published:** 2023-05-19

**Authors:** Francisco J. Enguita, Ana Lúcia Leitão, John S. Mattick

**Affiliations:** 1 Instituto de Medicina Molecular João Lobo Antunes, Faculdade de Medicina, Universidade de Lisboa, Av. Prof. Egas Moniz, 1649-028 Lisboa, Portugal; 2 Department of Chemistry, NOVA School of Science and Technology, FCT NOVA, Universidade NOVA de Lisboa, 2829-516 Caparica, Portugal; 3 School of Biotechnology and Biomolecular Sciences, UNSW Sydney, Sydney, NSW 2052, Australia

The central role of RNA molecules in cell biology has been an expanding subject of study since the proposal of the “RNA world” hypothesis 60 years ago [1,2]. The intrinsic functional flexibility of RNA, harboring both sequence and structural information, is exploited by cells to establish complex regulatory networks that are the foundations of cell function and differentiation, especially during multicellular development [3]. The advent of high-throughput techniques for dynamic genome, epigenome, transcriptome, and epitranscriptome analyses has opened up new avenues of research based on the principles of systems biology, allowing for the detailed characterization of many cellular processes centered on RNA molecules, both small and large [3,4,5]. This Special Issue compiles different contributions to the field of RNA regulatory networks, integrating 20 different contributions, divided into 8 reviews and 12 research manuscripts, which add to the complex picture of the functional landscape of RNA molecules in cell biology and human disease.

Two review papers included in this Special Issue are resources for analysis and methods to study the biological roles of RNA molecules. The manuscript by Mathlin et al. discusses the current knowledge of epitranscriptomic marks and proposes a categorization schema based on the reference ribonucleotide and its rounds of modifications (“stages”) until reaching the given modified form [5]. This constitutes a new approach for the investigation of epitranscriptomic dynamics in gene expression studies [5]. In parallel, Lee proposes a detailed guide for the functional analysis of non-coding RNAs (ncRNAs), starting from their characterization at the genomic level and describing a rational flowchart for their experimental dissection [6]. This paper also presents a critical discussion of the laboratory protocols used for the manipulation of ncRNA expression (overexpression and knock-down) and to determine the correct biological conclusions extracted from the data, showing the case of nc886 ncRNA as a working model [6].

Another review paper by Marasca et al. analyzes an oft-neglected topic in the field of RNA regulatory networks: the function of transposable genomic elements and their roles in cell physiology [7]. Transposable elements (TEs), covering approximately 45% of the human genome, are considered at present to be essential drivers of genome evolution [8]. TEs and the RNAs transcribed from them are mediators not only of multi-layered transcriptional regulatory functions in specific cells but also the regulation of cellular flexibility and adaptation to the environment [9]. The paper describes current methods for the study of TEs and how they can be used as potential therapeutic targets in human disease [7].

Mitochondrial RNAs are also discussed in this Special Issue. The article by Magistrati et al. focuses on how nucleotide modifications in mitochondrial rRNAs and tRNAs can be causative factors for mitochondrial diseases, referred to as modopathies, and how mutations in RNA-modifying enzymes can impair the efficiency of mitochondrial protein biosynthesis [10].

Among the various families of small ncRNAs, microRNAs (miRNAs) are the most studied group since their discovery in model organisms. These promiscuous negative post-transcriptional regulators play central roles in cell and developmental biology and human disease. Two of the review papers included in this Special Issue describe the role of miRNAs in the regulation of proteostasis and protein aggregation during brain and skeletal muscle aging [11] and how miRNAs act together with interferons to modulate antiviral responses in mammalian cells [12]. Complementing these reviews, the manuscript by Blasiak et al. explores an interesting metabolic network involving long non-coding RNAs (lncRNAs) [13]. In this paper, the authors describe the pleiotropic effect of dietary vitamin D on the maintenance of a normal epigenetic profile, linked to a lncRNA-centered regulatory network that may induce protective effects in some tumors [13].

Another article describes an alternative explanation of the effect exerted by myostatin in the control of muscle growth in mammals [14]. Using muscle cell lines and gene knock-out by CRISPR-Cas9, Huang et al. demonstrated that complete myostatin knock-out upregulates seven miRNAs, resulting in 28 downregulated genes, including TGFB1, FOS, and RB1, which are associated with tumorigenesis and cell proliferation, suggesting that myostatin may control muscle cell proliferation via the activation of miRNAs [14].

RNAs can also be dynamically interchanged between cells. Long-distance regulatory signals mediated by RNAs are often linked with their transport by vesicular conveyors. The RNA regulatory language is especially interesting when analyzing interspecies communication within holobionts involved in symbiotic or parasitic relationships, as discussed in the review paper by Leitão et al. [15].

The plasticity of RNA function is achieved by a smart combination of structure, sequence, and nucleotide modifications. The structural features of RNA molecules are clearly related to their functions and can be used by cells to modulate biological functions, scaffold macromolecular complexes, and localize RNAs to specific cellular compartments. Two papers included in the Special Issue describe the role of G-quadruplex structures in the function of coding and non-coding RNAs. Niu et al. show experimental evidence of the presence of a G-quadruplex structure in the internal ribosome entry site (IRES) of the VEGFA gene transcript [16]. In a cell model, the G-quadruplex stabilizer pyridostatin is reported to increase IRES activity, mediated by the G-quadruplex binding protein RBM4 [16]. Another paper by Imperatore et al. characterizes a curious G-quadruplex structure formed in a miRNA precursor containing an Alzheimer-associated genomic variant [17]. The existence of rs2291418 located in the precursor region of miRNA-1229 induces the formation of a G-quadruplex structure that establishes an equilibrium with the canonical hairpin pre-miRNA structure. The chemical equilibrium between both conformations is shown to be a crucial factor that controls the production of mature miR-1229-3p in Alzheimer’s patients and a potential therapeutic target for this disease [17].

The functional plasticity of ncRNAs is also exemplified by two experimental papers showing the function of lncRNAs in different biological contexts. Fefilova et al. showed that the murine lncRNA Morrbid is a key player in the regulation of the splicing of the proto-oncogene NRAS (neuroblastoma RAS viral oncogene homolog) in hepatocytes [18]. The alternative splicing of NRAS generates isoforms that are processed in some cases by nonsense-mediated decay, with the lncRNA contributing to mRNA quality control [18]. Another article describes the biological roles of a member of the subfamily of long-exonic non-coding RNAs (lencRNAs), PRKDC-210, which forms a complex with MED12, a component of the CDK8 complex, and is involved in the positive transcriptional regulation of several coding genes [19].

The interface between RNA-binding proteins (RBPs) and specific RNA molecules is central to the formation of RNA–protein complexes and condensates [20]. The role of one family of RBPs, the RNA helicases, was studied by Lodola et al. in the context of SARS-CoV-2 infection [21]. The authors show that human DDX1 and DDX3X helicases can interact with the viral nucleocapsid protein Np and increase its affinity for double-stranded RNA two- to four-fold in a helicase-independent manner. This interaction constitutes a host signal and may contribute to the establishment of SARS-CoV-2 infection in human cells [21]. Turbant et al. present a study of another RBP, the bacterial master riboregulatory protein Hfq [22], which is involved in several general aspects of bacterial RNA metabolism, including the regulation of transcriptional efficiency and RNA stability. The authors discovered that this pleiotropic regulator is also able to interact with membranes, controlling their integrity. The interaction is processed in an amyloid-like fashion, due to the oligomerization of the Hfq protein [22]. An additional paper by Chen et al. used transcriptomic data to characterize an RNA regulatory network centered on the role of RBP IGF2BP1 in adipocyte proliferation and differentiation in chickens, indicating the importance of the IGF2BP1 network in adipogenesis and fat metabolism [23].

Methods for the laboratory analysis of RNAs and their rigorous quality control are essential for the establishment of a solid knowledge base and are especially important in clinical applications. The article by Zhelankin et al. discusses the influence of pre-analytical factors in the profiling of circulating miRNAs [24]. Using small RNAseq, the authors describe a comprehensive assessment of the impact of the type of anticoagulant present in blood collection tubes on plasma miRNA profiles, proposing a standardized protocol that minimizes RNA loss and interference [24]. Similarly, the refinement of existing technologies to increase sensitivity allowed Lambert et al. to characterize a new class of small regulatory RNAs, designated as dodecaRNAs (doRNAs) [25]. These unusually short RNAs, which map to 5.8S rRNA, contain 12 nucleotides, are mainly cytoplasmic, and interact with the heterogeneous nuclear ribonucleoproteins (hnRNPs) A0, A1, and A2B1 but not with the RNA interference protein Argonaute 2. The authors show that the levels of doRNAs increase in conditions such as prostate cancer, suggesting their involvement in cell migration processes [25].

This Special Issue is concluded by two experimental papers describing how DNA methylation can be a source of variability in the expression of rRNA genes [26] and how codon bias can be a triggering factor for the accumulation of specific mRNAs into stress bodies in yeast [27]. Both articles are prime examples of how genomic variability can alter the structure and function of RNA regulatory networks.

Finally, we would like to acknowledge the serious, technically sound, and solid work presented by all of the authors who contributed to this Special Issue, together with the timely and constructive comments provided by all of the anonymous reviewers. We are in debt to you all, and we sincerely hope that we have the opportunity to collaborate with you again as we continue to explore the central role of RNA in cell biology, developmental biology, brain function, and transgenerational inheritance.
